# Identification and Evaluation of Colour Change in Rosemary and Biluochun Tea Infusions

**DOI:** 10.3390/metabo15040265

**Published:** 2025-04-11

**Authors:** Yuan Yuan, Caochuang Fang, Chaohan Li, Jiaqi You, Kun Ma

**Affiliations:** Shanghai Key Lab of Protected Horticultural Technology, Protected Horticultural Research Institute, Shanghai Academy of Agricultural Sciences, Shanghai 201106, China; yuan_23@saas.sh.cn (Y.Y.); biochzeu@saas.sh.cn (C.F.); lch505@163.com (C.L.); youjiaqi@saas.sh.cn (J.Y.)

**Keywords:** Biluochun, rosemary, colour of tea infusion, pink

## Abstract

Background: The colour of tea beverages during processing and storage significantly influences their visual quality. However, natural pink tea products are rare. This study investigated the mechanism behind the pink colouration in the mixed infusion of Biluochun (a green tea) and rosemary. Methods: Infusions of Biluochun (B), rosemary (R), and their mixture (BR), brewed with boiling water for 10 min, were analysed using liquid chromatography-mass spectrometry (LC-MS). Additionally, the pH value and tea pigment content were measured. Results: A total of 134 differential metabolites (DEMs) were detected. Kyoto Encyclopedia of Genes and Genomes (KEGG) analysis showed that phenylalanine metabolism and tyrosine metabolism pathways were enriched with abundant DEMs. Some amino acids in BR showed degradation. The content of pelargonin, a compound in the anthocyanin biosynthetic pathway, was significantly elevated in BR compared to that in B and R. DEMs related to fatty acid metabolism were at low levels in BR. Other compounds, such as quercetin, caffeate, rosmarinic acid, and isoferulic acid, were also more abundant in BR. No significant differences in pH value and tea pigment content were found among the three infusions. Conclusions: A model of pink colouration formation in BR was proposed based on the results of this study. Some substances in Biluochun and rosemary were released during the brewing process. Tyrosine was converted into p-coumaric acid, which further reacted to form pelargonin. Pelargonin, an orange-red (pH ≈ 5.0) anthocyanin, was the primary contributor to the pink colouration in BR. Additionally, p-coumaric acid formed co-pigments such as quercetin, caffeic acid, rosmarinic acid, and isovaleric acid. These co-pigments stabilised or enhanced the colour of pelargonin through co-pigmentation. The findings provide a theoretical basis for optimising tea processing techniques and improving quality control in beverage production.

## 1. Introduction

The tea plant (*Camellia sinensis*) is a perennial woody plant [1]. Its tender leaves can be used to process tea. Tea is usually categorised into three basic types based on the extent of fermentation: green tea (non-fermented), oolong tea (semi-fermented), and black tea (fully fermented) [2,3]. Different extents of fermentation result in different colours of brewed tea. Green tea produces a greenish or yellowish–green infusion, oolong tea yields a reddish–brown infusion, and black tea produces a dark brown infusion [2]. Catechins are important compounds affecting the colour changes in green tea [4]. Although most catechins are colourless, they change colour from red to brown upon oxidation [5]. The oxidation pathway of catechins occurs in the following sequence: catechin → theaflavin → thearubigin → theabrownin [6,7]. In green tea processing, the fixation process can inactivate enzymes, such as polyphenol oxidase and peroxidase, which are responsible for oxidising catechins. Consequently, most catechins in fresh leaves are retained. A small portion of catechins may undergo isomerisation and esterification due to heat [8]. In oolong tea processing, the withering process induces the localised oxidation of catechins at the leaf edges, resulting in the formation of certain oxidation products, such as bisflavanols, theaflavins, and thearubigins [8]. During black tea processing, the rolling and fermentation processes fully oxidise catechins and result in the formation of new compounds, such as theaflavins and thearubigins [8,9]. These compounds are critical to black tea quality and can be further oxidised and polymerised to form theabrownins [10]. Theaflavins exhibit an orange–red colour, thearubigins present a red–brown colouration, and theabrownins appear dark brown [11]. Furthermore, anthocyanins in tea infusions change colour based on pH, shifting from red under acidic conditions to colourless under neutral conditions or blue under alkaline conditions [5]. Chlorophyll, which is insoluble in water, decomposes and fades when heated. Under acidic environments, hydrogen ions replace magnesium ions in chlorophyll, producing brown pheophytin and altering the colour of tea infusions [5].

**Figure 1:**
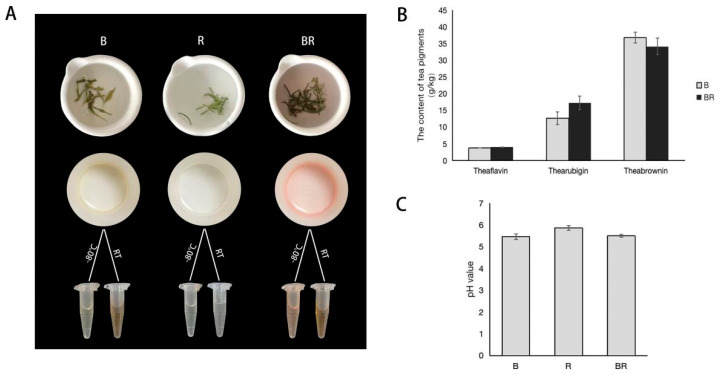
Colours of tea infusions, content of their tea pigment, and pH value. (**A**) Colours of three tea infusions after brewing for 10 min: B, Biluochun infusion; R, rosemary infusion; BR, tea infusion brewed with a mixture of Biluochun and rosemary; −80 °C, stored in a −80 °C freezer for 1 week; RT, stored at room temperature for 1 week. (**B**) The content of tea pigment. (**C**) The detection results of pH value.

Biluochun is a distinctive variety of green tea from China, with a history spanning nearly 1000 years. Its tea infusion is characterised by a yellowish–green colour and contains bioactive compounds associated with human health, such as polyphenols, polysaccharides, and flavonoids [12]. Rosemary (*Rosmarinus officinalis*), a perennial herb from the Labiaceae family, is also known as ocean dew or tansy [13]. It contains flavonoids, terpenes, sterols, and phenolic acids, endowing it with antibacterial, antioxidant, and anti-inflammatory properties, along with potential effects against tumours and immune regulation [14,15]. After brewing with boiling water, rosemary produces a clear infusion. Interestingly, when rosemary was brewed together with Biluochun for 10 min, the resulting tea infusion exhibited a pink colour (Figure 1A). In this study, the metabolites were analysed in three types of tea infusions (Biluochun, rosemary, and their mixture) to investigate the mechanisms of pink colouration in the mixed infusion of Biluochun and rosemary.

## 2. Materials and Methods

### 2.1. Preparation of Tea Infusions

Both 1.2 g of rosemary leaves and 1.2 g of Biluochun (tea leaves) were brewed with 300 mL of boiling water for 10 min. The weight ratio of rosemary leaves and Biluochun was 1:1. Then, the tea infusion was collected and recorded as BR. Only 1.2 g of Biluochun was brewed with 300 mL of boiling water for 10 min. Then, the tea infusion was collected and recorded as B. Only 1.2 g of rosemary leaves was brewed with 300 mL of boiling water for 10 min. Then, the tea infusion was collected and recorded as R. Biluochun (produced by Suzhou Xianling Tea Industry, Suzhou, China) was purchased from the supermarket. Its production date was 6 October, 2019. It was stored at −80 °C at the Shanghai Academy of Agricultural Sciences. Rosemary leaves (fresh sample) were collected from the Zhuanghuang Experimental Station of Shanghai Academy of Agricultural Sciences in spring.

### 2.2. Determination of the Total Contents of Theabrownin, Theaflavin and Thearubigin

The total contents of theabrownin, theaflavin, and thearubigin were analysed as described previously by Wang et al. [16]. The tea infusions were allowed to cool to room temperature before detection. A total of 50 mL of each tea infusion was mixed with 50 mL of ethyl acetate (EtOAc) for 5 min, after which the solution formed aqueous and EtOAc layers. The following solutions were prepared from these layers. Solution A: 4 mL of the EtOAc layer was diluted to 25 mL with 95% ethanol. Solution B: 25 mL of the tea infusion was shaken with 25 mL of butyl alcohol for 3 min, after which the layers were separated. Then, 2 mL of the aqueous layer was diluted to 25 mL with 2 mL of saturated oxalic acid solution, 6 mL of distilled water, and 15 mL of 95% ethanol. Solution C: 25 mL of the EtOAc layer was mixed with 25 mL of NaHCO_3_ solution (2.5 g/100 mL) for 30 s. After settling, the upper layer was discarded. Then, 4 mL of the lower layer was diluted to 25 mL with 95% ethanol. Solution D: 2 mL of the aqueous layer was diluted to 25 mL with 6 mL of distilled water, 2 mL of saturated oxalic acid solution, and 15 mL of 95% ethanol. The optical densities of the four solutions (Solutions A–D) were measured at 380 nm and recorded as EA, EB, EC, and ED, respectively. The total concentration of tea browning, theaflavins, and thearubigins was determined using the formula of Wang et al. [16].

TFs = EC × 2.25/dried weight(%) × 100%

TRs = 7.06 × (2EA + 2ED − EC − 2EB)/dried weight (%) × 100%

TBs = 2EB × 7.06/dried weight (%) × 100%

### 2.3. pH Value Detection

Approximately 30 mL of the tea infusion was placed in a 50 mL beaker. A pre-calibrated pH meter was inserted into the cooled tea infusion at room temperature. Each tea infusion was measured in triplicate to ensure biological repetition.

### 2.4. Metabolomics Analysis Using Liquid Chromatography–Mass Spectrometry (LC–MS)

The tea infusion samples (B, R, and BR) were sent to Nomi Metabolism Company for LC–MS analysis. Each sample included six biological replicates. The unprocessed MS files were converted into mzXML format using ProteoWizard. Peak recognition, filtering, and alignment in the LC–MS data were performed using XCMS software (1.42.0) [17]. All peak areas were then exported to Excel for batch normalisation. Then, these data underwent autoscaling and mean-centring and were scaled to unit variance before multivariate statistical analyses, which included principal component analysis (PCA) utilising SIMCA-P 13.0. Variables with variable importance in projection (VIP) values of >1 and *p*-values of <0.05 were considered significantly different. Tandem MS spectra were crucial for identifying the metabolites by comparing them with authentic samples and datasets. The databases used for annotation included the Human Metabolome Database, Metlin, and LipidMap. The heatmaps and volcano plot were analysed and generated using online tools (https://www.genescloud.cn/chart/ChartOverview (accessed on 20 September 2024)).

## 3. Results

### 3.1. Identification of the Colour, Tea Pigment, and pH Value of the Infusions

After brewing Biluochun, rosemary, and their mixture with boiling water for 10 min, the three tea infusions displayed distinct colours. The Biluochun tea infusion (B) was light yellow, the rosemary tea infusion (R) was colourless, and their mixture (BR) appeared pink (Figure 1A).

Tea pigment is a water-soluble phenolic pigment produced by the oxidative polymerisation of tea polyphenols in tea plants [18]. It is related to the colour of the tea infusion, including theaflavins, thearubigins, and theabrownins [16]. In this study, theaflavins, thearubigins, and theabrownins were detected in B and BR. As shown in Figure 1B, the trends in the content of the three pigments in B and BR were similar: the theabrownin content was the highest, followed by thearubigins, whereas the theaflavin content was the lowest. However, there was no significant difference in the pigment content between B and BR. Results indicated that the tea pigment was not responsible for the pink colour observed in BR. Existing literature showed a correlation between the pH value and the colour of the tea infusion [19,20]. The pH values in B, R and BR (pH = ~5.5) showed no significant difference (Figure 1C), indicating that the pink colour in BR was not caused by changes in the pH value.

### 3.2. Metabolomic Analysis of the Tea Infusions

**Figure 2:**
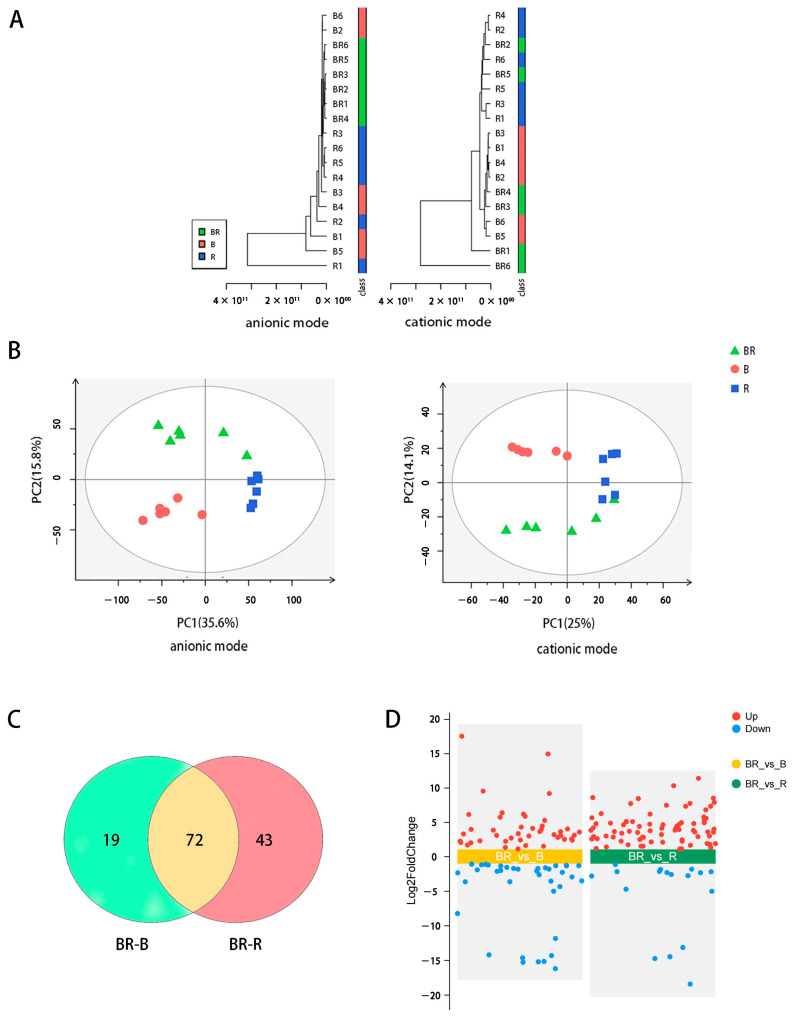
Metabolomic analysis of tea infusions. (**A**) The hierarchical cluster analysis in anion mode or cationic mode of B, R, and BR. (**B**) PCA score plot in anion mode or cationic mode of B, R, and BR. (**C**) A Venn plot of 134 differential metabolites (DEMs) (BR-B: metabolites were obtained by comparing BR and B metabolites, BR-R: metabolites were obtained by comparing BR and R metabolites). (**D**) A volcano plot of 134 DEMs in BR-B or BR-B.

To explore the reason for the pink colour in BR, LC–MS was used to analyse the metabolite profiles in B, R and BR. After data analysis, 4207 positive and 8865 negative ion variables were detected. Hierarchical cluster analysis classified B and R into two categories in the cationic mode but not in the anionic mode. In contrast, BR was separated from B and R in the anionic mode but not in the cationic mode (Figure 2A). The repeatability and differences in the samples can be analysed based on the dispersion degree shown in PCA to determine instrument stability and data validity [21]. Regardless of the positive or negative ion mode, the distribution points of the six biological replicates in the same sample were close to each other (Figure 2B). This indicated that the signal detection was stable and the data were reliable. The distance between the distribution positions of B and BR was greater than that between R and BR in PCA (Figure 2B), indicating that there were a higher number of differentially expressed metabolites (DEMs) between B and BR.

A total of 205 DEMs were obtained by comparing BR and R samples (BR-R) and BR and B samples (BR-B) separately (*p* ≤ 0.05, VIP ≥ 1, |log_2_FoldChange| ≥ 1). It is worth noting that some metabolites in B and R samples, which were less prone to chemical reactions, were also present in BR samples, as BR was a mixed tea infusion of B and R. These inert metabolites might be classified into DEMs when comparing the difference in metabolites among different samples. For example, an inert metabolite, α, was present in B but not in R. It meant that α also existed in BR and became a DEM when comparing BR and R (BR-R). However, α did not contribute to the change in the colour of BR. To eliminate potential misidentifications of DEMs, these 205 metabolites were further screened according to the following condition: FoldChange (BR-R)/FoldChange (B-R) > 1.3 or <0.7, and FoldChange (BR-R)/FoldChange (B-R) >1.3 or <0.7. Finally, a total of 134 DEMs were obtained (Table S1) and used to construct a Venn plot and a Volcano plot. Among them, 72 were detected in BR-R and BR-B samples. Overall, 43 and 19 of the remaining 62 DEMs were specific to BR-R and BR-B samples, respectively (Figure 2C,D).

**Figure 3:**
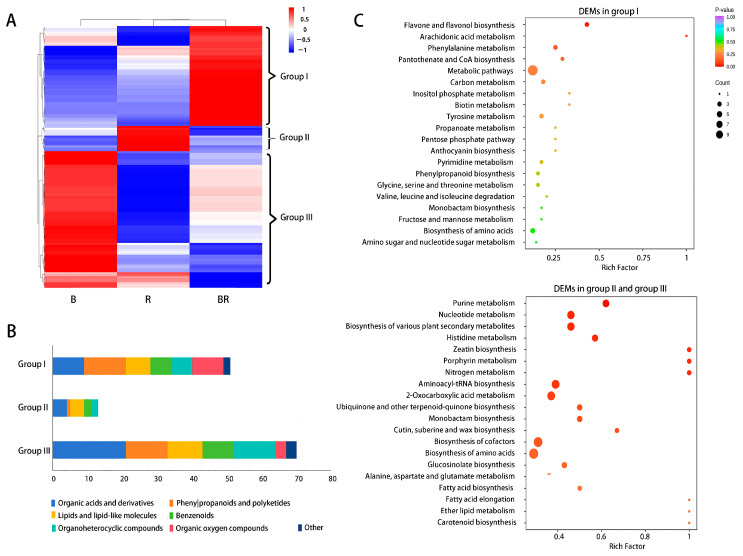
Clustering, classification, and enrichment analysis of 134 DEMs. (**A**) Heatmap analysis of 134 DEMs (blue: low content, red: high content), (**B**) The chemical taxonomy of 134 DEMs, (**C**) The Kyoto Encyclopedia of Genes and Genomes (KEGG) enrichment analysis for 134 DEMs.

A heatmap analysis was used to visualise the changes in these critical metabolites among B, R, and BR samples, with a colour gradient from blue (low content) to red (high content) (Figure 3A). Cluster analysis categorised the 134 DEMs into 3 groups: (I) DEM content was highest in BR compared with that in B and R, (II) DEM content was highest in R compared with that in B and BR, (III) DEM content was highest in B compared with that in R and BR, and DEM content was lowest in BR compared with that in B and R. It was speculated that the DEMs in B samples underwent more changes after being mixed with R samples. This was consistent with the PCA results, which showed overlap between R and BR but not between B and BR. According to the chemical taxonomy of metabolites, 134 DEMs were classified into seven categories, including organic acids and derivatives (26.87%), phenylpropanoids and polyketide (18.66%), lipids and lipid-like molecules (15.67%), organoheterocyclic compounds (10.45%), and others (Figure 3B; Table S1).

**Figure 4:**
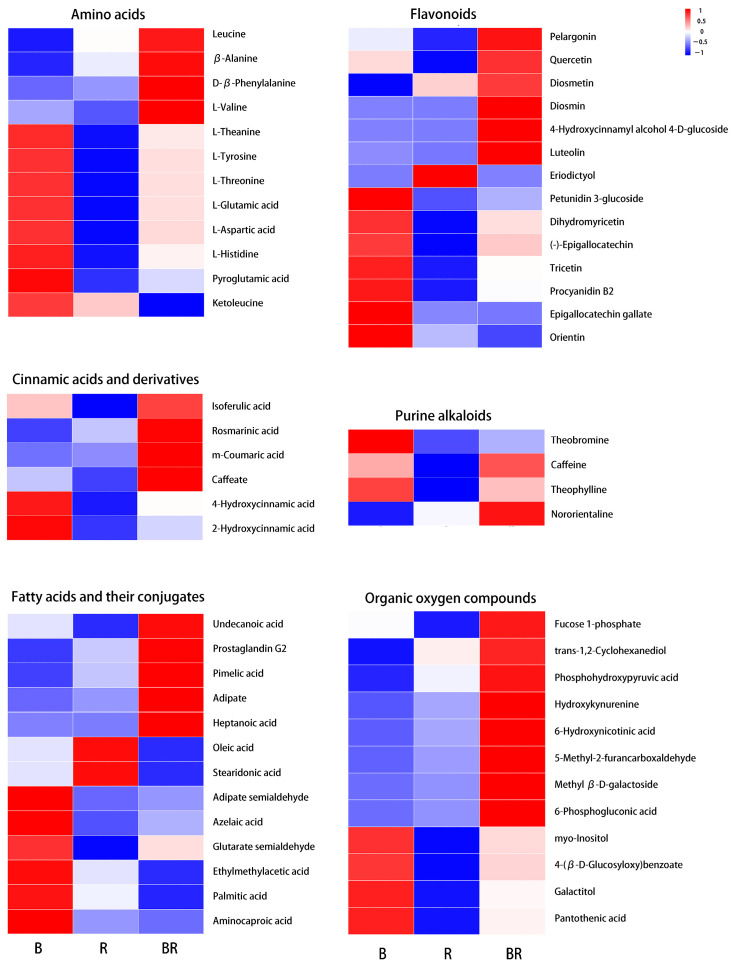
Heatmap of major DEMs (blue: low content, red: high content).

**Organic acids and derivatives:** Amino acids, a key group of metabolites, significantly contribute to the taste of the tea infusions [22]. Within the category of organic acids and derivatives, 12 amino acids were included. The contents of L-valine, β-alanine, leucine, and D-β-phenylalanine were higher in BR than in either B or R, whereas the levels of L-histidine, L-threonine, L-aspartic acid, L-tyrosine, L-theanine, pyroglutamic acid, and L-glutamic acid were lower in BR than in B (Figure 4, Table S1). Notably, the ketoleucine content was lower in BR than that in both B and R. D-β-phenylalanine, a key pharmaceutical building block, is not common in plants This suggested that when Biluochun and rosemary were mixed and brewed, some new amino acids were formed, but a larger proportion underwent degradation. According to the Kyoto Encyclopedia of Genes and Genomes (KEGG) enrichment analysis (Figure 3C), DEMs with high content only in BR were enriched in pathways such as phenylalanine metabolism; tyrosine metabolism; propanoate metabolism; glycine, serine and threonine metabolism; and valine, leucine, and isoleucine degradation. Among these, the phenylalanine metabolism and tyrosine metabolism pathways were considered to play significant roles in the Biluochun–rosemary fusion, as they had numerous enriched DEMs with smaller *p*-values (Figure 3C).

**Phenylpropanoids and polyketides:** Phenylpropanoids are a class of organic compounds featuring benzene rings and propionic acid groups, widely distributed in nature and known for their important biological activities. Plants can synthesise phenylpropanoids through phenylalanine metabolism and tyrosine metabolism pathways [23]. Among the 25 DEMs of phenylpropanoids, 14 were flavonoids and 6 were cinnamic acid and its derivatives (Figure 4, Table S1). The KEGG enrichment analysis showed that there were three metabolic pathways associated with flavonoids (Figure 3C). The DEMs content enriched in flavone/flavonol biosynthesis and anthocyanin biosynthesis was higher in BR than in B and R, whereas that enriched in carotenoid biosynthesis was lower in BR than in B or R. Notably, a high content of pelargonin, an anthocyanin, was detected in BR. Anthocyanins are typically associated with the colouration of plants [24]. Therefore, it was speculated that pelargonin was an important metabolite that influences the colour change in BR. In addition to anthocyanins, flavonoids, phenolic acids, and organic acids have been reported to contribute to colour changes, as they can interact with anthocyanins to produce pigmentation [25]. Based on the classification, a high content of five non-anthocyanin flavonoids (luteolin 7-O-glucuronide, luteolin, quercetin, diosmin, and rosmarinic acid) was detected in BR, which might engage in co-pigmentation with pelargonin.

Catechin compounds are the main bioactive components in tea, including catechin, epicatechin, gallocatechin, epigallocatechin (EGC), catechin gallate, epicatechin gallate, gallocatechin gallate, and epigallocatechin gallate (EGCG). There was no significant difference in the levels of most catechin compounds between B and BR, except for EGC and EGCG, which were found to be lower in BR than in B (Table S1). However, it remains unclear whether these two compounds contribute to the colour difference between BR and B.

**Lipids and lipid-like molecules:** Lipids and lipid-like molecules are essential components of biological membranes, energy storage molecules, signalling molecules, and other cellular structures [26,27]. Additionally, lipids are a major source of aroma in tea. In this study, 13 fatty acids and their conjugates as well as 4 prenol lipids were detected (Figure 4, Table S1). KEGG enrichment analysis revealed that DEMs enriched in fatty acid-related metabolic pathways were present at low levels in BR (Figure 3C). The decrease in fatty acids, combined with the metabolic processes of flavonoids/anthocyanins, porphyrins, chlorophyll, carotenoids, and steroids, collectively leads to the alteration in the colour of tea leaves [28]. Therefore, the low fatty acid content might contribute to colour changes in BR.

**Others:** Purine alkaloids in tea, such as caffeine, theobromine, and theophylline, are methyl derivatives of xanthine [29]. In this study, caffeine and theophylline showed no significant difference between B and BR, and theobromine content was lower in BR than in B (Figure 4, Table S1). Nororientaline, an isoquinoline alkaloid, was present at higher levels in BR(Figure 4, Table S1). Additionally, 8 of the 12 organic oxygen compounds had higher content in BR, and these metabolites were primarily involved in amino acid and carbohydrate metabolism (Figure 4, Table S1).

## 4. Discussion

**Figure 5:**
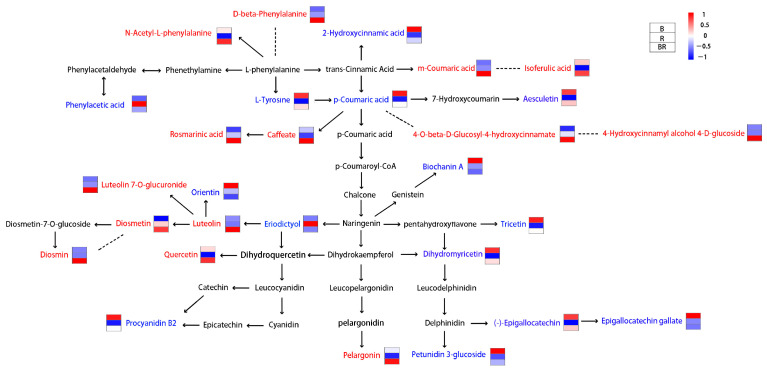
Metabolic pathway of flavonoids (the box represents the heatmap of mean values of B, R, and BR in the LC-MS, blue: low content, red: high content).

It was interesting that the colour of the infusion changed when Biluochun and rosemary were mixed and brewed (Figure 1A). The colour of tea and its infusion is primarily influenced by pH, anthocyanins, flavonoids, and tea pigment [3,30,31]. In this study, the pink colouration observed in BR was not associated with tea pigment or pH (Figure 1B,C). Studies have indicated that the vibrant colours of plants are primarily linked to three types of compounds: anthocyanins [32], carotenoids [33], and betalains [34]. Numerous studies have reported that the red, blue, purple, and transitional colours of plants are attributed to anthocyanins, whereas the yellow and orange colours of plants, along with their transition colourations, are primarily attributed to carotenoids [32,33,35]. Betalains are the yellow and violet pigments found exclusively in Caryophyllales (including cactus and amaranth), which never co-occur with anthocyanins in plants [34,35]. According to the KEGG pathway analysis, only (S)-abscisic acid with low content in BR was enriched in carotenoid biosynthesis. No DEMs were enriched in betalain biosynthesis (Figure 3C), indicating that carotenoids and betalains did not contribute to the colour change in BR. The metabolic pathway involved in anthocyanin synthesis has been explored. Phenylalanine is converted into p-coumaric acid via phenylpropanoid biosynthesis, whereas tyrosine is metabolised through tyrosine metabolism to produce p-coumaric acid. p-Coumaric acid undergoes a series of enzymatic reactions to form dihydroflavonol. These precursor compounds are further modified by enzymes, ultimately resulting in anthocyanins with distinct colours (Figure 5) [36,37,38]. In plants, anthocyanidins primarily exist as glycosylated or acylated derivatives [35]. Pelargonin, a phytochemical by glycosylation of pelargonidin [39], was the only anthocyanin in the DEMs. Its content in BR was significantly higher than that in B and R (Figure 5). Yuan et al. found that pelargonin was the sole anthocyanin species in purple-heart radish [39]. Due to the glycosylation, pelargonin exhibited higher stability in different pH, temperature, light, and metal ions environments than that of pelargonidin and obviously presented a darker colour (orange-red, pH ≈ 5.0) than pelargonidin [39]. Red and pink roses obtain their colour from pelargonin [40]. Strawberries and red radish peels are the main sources of pelargonins’ extraction [41]. It has also been detected in some special tea trees, such as Zijuan and Ziyan, whose tea leaves are reddish–purple [42,43]. However, there are few reports in green tea. Therefore, pelargonin is likely one of the important factors influencing the formation of pink colour in BR.

Besides anthocyanins, carotenoids and beet pigments, flavonoids (non-anthocyanins), phenolic acids, organic acids, and other substances also contribute to colour and play a role in co-pigmentation. Co-pigmentation refers to the formation of non-covalent complexes between anthocyanins and co-pigment compounds [25,44]. These complexes prevent water molecules from nucleophilically attacking anthocyanins, thus inhibiting the formation of chalcone structures and protecting anthocyanins from degradation [45]. Co-pigments such as quercetin, caffeic acid (caffeate), ferulic acid, gallic acid, protocatechuic acid, and rosmarinic acid have been shown to be efficient phenolic co-pigments and are applied to enhance and/or stabilise anthocyanin colour [45,46,47,48]. The levels of quercetin, caffeate (caffeic acid, C01197), rosmarinic acid, and isoferulic acid (an isomeric form of ferulic acid) in BR were higher than those in B or R (Figure 5; Table S1). These substances were likely to play a role in stabilising and enhancing the pink colour in BR. Sevcan Erşan [49] discovered that flavonoids luteolin and orientin were effective co-pigments for pelargonidin 3-glucoside. In this study, compared to B and R, luteolin was present at higher levels in BR, whereas orientin was found at lower levels in BR (Figure 5; Table S1). Therefore, luteolin may play a more significant role in the pink colour exhibited in BR compared to orientin.

Among the metabolites of luteolin, a particular compound caught our attention: diosmin. Diosmin (diosmetin 7-O-rutinoside), a flavone glycoside of diosmetin, is a flavonoid glycoside found in citrus fruit peels and is commonly used as a phlebotomic, non-prescription dietary supplement for treating haemorrhoids or chronic venous diseases [50]. There are limited reports on diosmin in tea. Yinai Deng et al. [51] found that diosmin can be synthesised from luteolin via a multienzyme cascade reaction. The methyl group is preferentially transferred to the 4′-hydroxy group of luteolin, which has adjacent substituents, forming diosmetin. Diosmetin is a key intermediate in diosmin synthesis, and CiUGT11 (UGT88B3) specifically converts diosmetin to diosmetin 7-O-glucoside, leading to the formation of diosmin. Interestingly, these metabolomics data showed that diosmetin 7-O-glucoside was not detected, but the important intermediate diosmetin was detected, with a high content observed only in BR (Figure 5; Table S1). Moreover, the luteolin and luteolin 7-O-glucoside contents were high in BR (Figure 5; Table S1). Luteolin was detected in various tea plants [3,11,52]. These results suggested that diosmin was generated by the reaction of diosmetin when B and R were mixed but may not be generated via diosmetin 7-O-glucoside.

During the processing and storage of tea beverages, changes in colour are key factors affecting the visual quality of tea beverages. Natural pink tea beverages are rarely observed in the market, and the findings of this study provide theoretical basis for the preparation of natural pink tea beverages. However, tea beverages are extremely unstable under heat. During storage of B, R and BR at room temperature for 1 week, the colour of B gradually became similar to that of BR (Figure 1A). Further research is warranted to explore how to slow down or prevent colour changes in tea infusions.

## 5. Conclusions

**Figure 6:**
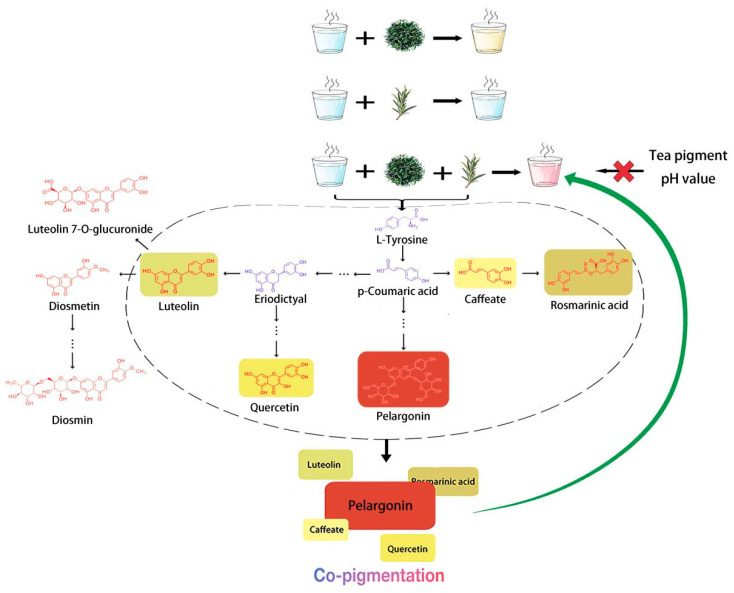
A model of pink colouration formation in the infusion of brewed Biluochun and rosemary (The pictures of Biluochun, rosemary, and cups were generated by Doubao (AI), https://www.doubao.com/chat/, accessed on 25 February 2025).

Based on the above results, a model has been developed to explain the pink colour produced after brewing Biluochun tea and rosemary for 10 min (Figure 6). High-temperature water acted on Biluochun and rosemary, causing the release of precursor substances, such as L-tyrosine, from within the cells. This amino acid was converted into p-coumaric acid. p-Coumaric acid then underwent further reactions, enzymatic reaction or chemical reaction, to form pelargonin. Pelargonin is an orange-red anthocyanin pigment associated with the pink and red colours observed in plants. p-Coumaric acid not only served as a key precursor in pigment synthesis but also generated flavonoids such as quercetin, caffeic acid, rosmarinic acid, and isovaleric acid. These compounds were co-pigments that stabilised or enhanced the colour of pelargonin through co-pigmentation. The results offer a theoretical foundation for refining tea processing methods and enhancing quality control in beverage manufacturing.

## Data Availability

Raw data was deposited in National Genomics Data Center (https://www.cncb.ac.cn/, accessed on 12 March 2025) under the BioProject: PRJCA037247.

## Supplementary Materials

The following supporting information can be downloaded at: https://www.mdpi.com/article/10.3390/metabo15040265/s1, Table S1 The information of 134 differential metabolites.
