# Supplementary material for: Identification and Evaluation of Colour Change in Rosemary and Biluochun Tea Infusions
Source: Metabolites. 2025 Apr 11;15(4):265. doi: 10.3390/metabo15040265 (PMC12029317; doi:10.3390/metabo15040265)
Supplement: Supplementary file 1 [file metabolites-15-00265-s001.zip › metabolites-3553564-supplementary.pdf]

Supplementary Table

Table S1 The information of 134 differential metabolites

Number	Name	KEGG	log2(FC_BR/R)	log2(FC_BR/B)	Superpathway	Superclass	class	sub_class
1	Ketoleucine	C00233	-14.788	-15.327	Amino acid	Organic acids and derivatives	Keto acids and derivatives	Short-chain keto acids and derivatives
2	Methyl benzoate	C20645	-14.514	-14.683	Unknow	Benzenoids	NULL	NULL
3	(R)-5,6-Dihydrothymine	C21028	-13.184	-14.361	Nucleotide	NULL	NULL	NULL
4	Oleic acid	C00712	-4.7567	-3.5388	Lipid	Lipids and lipid-like molecules	Fatty Acyls	Fatty acids and conjugates
5	Stearidonic acid	C16300	-2.8449	-1.8243	Lipid	Lipids and lipid-like molecules	Fatty Acyls	Lineolic acids and derivatives
6	2-Methoxyestrone	C05299	-2.787	3.9831	Lipid	Lipids and lipid-like molecules	Steroids and steroid derivatives	Estrane steroids
7	5-Valerolactone	C02240	-2.6162	-5.0559	Lipid	NULL	NULL	NULL
8	5-Hydroxymethyluracil	C03088	-2.3551	-1.621	Unknow	Organoheterocyclic compounds	Diazines	Pyrimidines and pyrimidine derivatives
9	8-Amino-7-oxononanoate	C01092	-2.3255	-3.6629	Cofactors and Vitamins	Lipids and lipid-like molecules	NULL	NULL
10	Neopterin	C05926	-2.2105	0.49003	Cofactors and Vitamins	Organoheterocyclic compounds	Pteridines and derivatives	Pterins and derivatives
11	(+)-Camphor	C00808	-2.1768	-2.2605	Lipid	Lipids and lipid-like molecules	NULL	NULL
12	Phenylacetic acid	C07086	-1.8684	0.89178	Amino acid	Benzenoids	Benzene and substituted derivatives	NULL

13	Aminoadipic acid	C00956	-1.8383	-1.0236	Amino acid	Organic acids and derivatives	Carboxylic acids and derivatives	Amino acids, peptides, and analogues
14	Creatine	C00300	-1.1588	0.55503	Amino acid	Organic acids and derivatives	Carboxylic acids and derivatives	Amino acids, peptides, and analogues
15	Undecanoic acid	C17715	1.0937	0.54207	Lipid	Lipids and lipid-like molecules	Fatty Acyls	Fatty acids and conjugates
16	3D-3,5/4-Trihydroxycyclohexane-1,2-dione	C04287	1.1262	1.639	Carbohydrate	NULL	NULL	NULL
17	6-Phosphogluconic acid	C00345	1.2768	1.463	Carbohydrate	Organic oxygen compounds	Organooxygen compounds	Carbohydrates and carbohydrate conjugates
18	Pantothenic acid	C00864	1.3672	-0.60984	Cofactors and Vitamins	Organic oxygen compounds	Alcohols and polyols	Polyols
19	2-Furoate	C01546	1.4742	-0.63941	Xenobiotics	Organoheterocyclic compounds？	Furans	Furoic acid and derivatives
20	L-Valine	C00183	1.4747	1.0721	Amino acid	Organic acids and derivatives	Carboxylic acids and derivatives	Amino acids, peptides, and analogues
21	D-beta-Phenylalanine	C20488	1.6031	2.0102	Amino acid	Organic acids and derivatives？	NULL	NULL
22	Phosphoglycolic acid	C00988	1.6745	2.9953	Carbohydrate	Organic acids and derivatives	Organic phosphoric acids and derivatives	Phosphate esters
23	Rosmarinic acid	C01850	1.7396	9.1463	Amino acid	Phenylpropanoids and polyketides	Cinnamic acids and derivatives	Hydroxycinnamic acids and derivatives
24	5-Methyl-2-furancarboxaldehyde	C11115	1.837	2.6492	Unknow	Organic oxygen compounds	Carbonyl compounds	Aldehydes
25	Methyl beta-D-galactoside	C03619	1.8626	2.2447	Unknow	Organic oxygen compounds ?	NULL	NULL

26	2-Hydroxycinnamic acid	C01772	2.0562	-1.2423	Unknow	Phenylpropanoids and polyketides	Benzene and substituted derivatives	1-hydroxy-2-unsubstitute d benzenoids
27	Catechol	C00090	2.0893	-1.1645	Xenobiotics	NULL	NULL	NULL
28	Tetrahydrodipicolinate	C03972	2.1891	0.64593	Amino acid	Organic acids and derivatives	Carboxylic acids and derivatives	Amino acids, peptides, and analogues
29	Galactitol	C01697	2.3976	-0.77034	Carbohydrate	Organic oxygen compounds	Organooxygen compounds	Carbohydrates and carbohydrate conjugates
30	N-Acetyl-L-phenylalanine	C03519	2.4439	0.59648	Amino acid	Organic acids and derivatives	Carboxylic acids and derivatives	Amino acids, peptides, and analogues
31	Hypoxanthine	C00262	2.523	-0.68503	Nucleotide	Organoheterocyclic compounds	Imidazopyrimidines	Purines and purine derivatives
32	6-Hydroxynicotinic acid	C01020	2.5607	17.495	Cofactors and Vitamins	Organic oxygen compounds	Pyridines and derivatives	Pyridinecarboxylic acids and derivatives
33	Adenine	C00147	2.761	-0.58779	Nucleotide	Organoheterocyclic compounds	Imidazopyrimidines	Purines and purine derivatives
34	4-Nitrophenol	C00870	2.791	0.53596	Xenobiotics	Benzenoids	Phenols	Nitrophenols
35	Styrene Oxide	C02083	2.7967	-1.7331	Unknow	Benzenoids	Benzene and substituted derivatives	Ethers
36	Vanillylmandelic acid	C05584	2.8587	5.8291	Amino acid	Benzenoids	Phenols	Methoxyphenols
37	p-Octopamine	C04227	2.8664	-0.98899	Unknow	Benzenoids	Phenols	1-hydroxy-2-unsubstitute d benzenoids
38	Gentisic acid	C00628	2.9504	2.3356	Amino acid	Benzenoids	Benzene and substituted derivatives	Benzoic acids and derivatives
39	L-2,4-diaminobutyric acid	C03283	2.9599	3.1599	Amino acid	Organic acids and derivatives	Carboxylic acids and derivatives	Amino acids, peptides, and analogues
40	Guanosine	C00387	3.1572	-0.54819	Nucleotide	Nucleosides,	Purine nucleosides	NULL

						nucleotides, and analogues		
41	Isoferulic acid	C10470	3.3566	0.4467	Xenobiotics	Phenylpropanoids and polyketides	Cinnamic acids and derivatives	Hydroxycinnamic acids and derivatives
42	D-Gulono-1,4-lactone	C05410	3.4623	3.6004	Unknow	Organoheterocyclic compounds	NULL	NULL
43	L-Histidine	C00135	3.507	-0.83437	Amino acid	Organic acids and derivatives	Carboxylic acids and derivatives	Amino acids, peptides, and analogues
44	Luteolin 7-O-glucuronide	C03515	3.5437	5.2277	Lipid	Phenylpropanoids and polyketides	Benzene and substituted derivatives	1-benzopyrans
45	myo-Inositol	C00137	3.5509	-0.63603	Carbohydrate	Organic oxygen compounds	Organooxygen compounds	Alcohols and polyols
46	4-Hydroxycinnamic acid	C00811	3.6024	-0.93409	Xenobiotics	Phenylpropanoids and polyketides	Cinnamic acids and derivatives	Hydroxycinnamic acids and derivatives
47	Quinate	C00296	3.6488	0.44644	Amino acid	NULL	NULL	NULL
48	N6-Acetyl-L-lysine	C02727	3.7154	-0.52555	Amino acid	Organic acids and derivatives	Carboxylic acids and derivatives	Amino acids, peptides, and analogues
49	Glutarate semialdehyde	C03273	3.7773	-0.65494	Amino acid	Lipids and lipid-like molecules	Fatty Acyls	Fatty acids and conjugates
50	m-Coumaric acid	C12621	3.7852	14.905	Amino acid	Phenylpropanoids and polyketides	Benzene and substituted derivatives	1-hydroxy-2-unsubstitute d benzenoids
51	4-Hydroxycinnamyl alcohol 4-D-glucoside	C05855	3.8107	3.8031	Unknow	NULL	NULL	NULL
52	Caffeate	C01197	3.8975	1.5661	Xenobiotics	NULL	NULL	NULL
53	L-Glutamine	C00064	3.9577	-0.53796	Amino acid	Organic acids and derivatives	Carboxylic acids and derivatives	Amino acids, peptides, and analogues

54	(S)-Abscisic acid	C06082	4.006	-0.68504	Lipid	Lipids and lipid-like molecules	Prenol lipids	Sesquiterpenoids
55	Gallic acid	C01424	4.0793	-1.2078	Xenobiotics	Benzenoids	Benzene and substituted derivatives	Benzoic acids and derivatives
56	Heptanoic acid	C17714	4.13	4.0698	Lipid	Lipids and lipid-like molecules	Fatty Acyls	Fatty acids and conjugates
57	8-Hydroxyquinoline	C19434	4.3542	-0.61566	Unknow	NULL	NULL	NULL
58	Hydroxyindole	C02040	4.3852	1.772	Unknow	NULL	NULL	NULL
59	Fucose 1-phosphate	C02985	4.5871	0.97859	Carbohydrate	Organic oxygen compounds	Organooxygen compounds	Carbohydrates and carbohydrate conjugates
60	L-Tryptophan	C00078	4.5902	-0.68569	Amino acid	Organoheterocyclic compounds	Indoles and derivatives	Indolyl carboxylic acids and derivatives
61	Pelargonin	C08725	4.6555	1.0917	Lipid	Phenylpropanoids and polyketides	Flavonoids	Flavonoid glycosides
62	1,3,7-Trimethyluric acid	C16361	4.6739	-1.8386	Xenobiotics	Organoheterocyclic compounds	Imidazopyrimidines	Purines and purine derivatives
63	dTMP	C00364	4.7491	-0.63453	Nucleotide	Nucleosides, nucleotides, and analogues	Pyrimidine nucleotides	Pyrimidine deoxyribonucleotides
64	Luteolin	C01514	4.7595	3.5275	Lipid	Phenylpropanoids and polyketides	Flavonoids	Flavones
65	L-Threonine	C00188	4.8344	-0.68589	Amino acid	Organic acids and derivatives	Carboxylic acids and derivatives	Amino acids, peptides, and analogues
66	Phenyl acetate	C00548	4.8828	2.0541	Unknow	Benzenoids	NULL	NULL
67	Glycerophosphocholine	C00670	4.8915	-1.2289	Lipid	Lipids and lipid-like molecules	Glycerophospholipids	Glycerophosphocholines

68	4-Acetamidobutanoic acid	C02946	5.0221	-1.1154	Amino acid	Organic acids and derivatives	Carboxylic acids and derivatives	Amino acids, peptides, and analogues
69	2-Aminoacrylic acid	C02218	5.0596	-0.6154	Amino acid	Organic acids and derivatives	Carboxylic acids and derivatives	Amino acids, peptides, and analogues
70	AMP	C00020	5.0707	-1.3711	Nucleotide	Nucleosides, nucleotides, and analogues	Purine nucleotides	Purine ribonucleotides
71	Tricetin	C10192	5.3602	-0.95021	Lipid	Phenylpropanoids and polyketides	Flavonoids	Flavones
72	Phenylpropanoate	C05629	5.5348	6.3437	Amino acid	NULL	Phenylpropanoic acids	NULL
73	L-Glutamic acid	C00025	5.6094	-0.69236	Amino acid	Organic acids and derivatives	Carboxylic acids and derivatives	Amino acids, peptides, and analogues
74	Quercetin	C00389	5.7199	0.65278	Lipid	Phenylpropanoids and polyketides	Flavonoids	Flavones
75	Thymidine	C00214	6.1411	0.50041	Nucleotide	Nucleosides, nucleotides, and analogues	Pyrimidine nucleosides	Pyrimidine 2'-deoxyribonucleosides
76	Aesculetin	C09263	6.2214	-0.51826	Unknow	Phenylpropanoids and polyketides	Coumarins and derivatives	Hydroxycoumarins
77	4-(beta-D-Glucosyloxy)benzoate	C03993	6.7624	-0.62664	Cofactors and Vitamins	?Organic oxygen compounds	Benzene and substituted derivatives	Alcohols and polyols
78	Petunidin 3-glucoside	C12139	7.2815	-2.1482	Lipid	Phenylpropanoids and polyketides	Flavonoids	Flavonoid glycosides
79	L-Aspartic acid	C00049	7.2889	-0.64645	Amino acid	Organic acids and derivatives	Carboxylic acids and derivatives	Amino acids, peptides, and analogues
80	Citric acid	C00158	7.4279	-0.61491	Carbohydrate	Organic acids and	Carboxylic acids and	Tricarboxylic acids and

						derivatives	derivatives	derivatives
81	S-[(Z)-N-Hydroxy(phenyl)acetimidoyl]-L-glutathione	C21622	7.6743	-0.538	Unknow	NULL	NULL	NULL
82	(-)-Epigallocatechin	C12136	7.7032	-0.55939	Lipid	Phenylpropanoids and polyketides	Flavonoids	Flavans
83	3,4-Dihydroxymandelic acid	C05580	7.8496	-0.70834	Amino acid	Benzenoids	Phenols	Benzenediols
84	Procyanidin B2	C17639	8.4039	-1.0133	Lipid	Phenylpropanoids and polyketides	Flavonoids	Biflavonoids and polyflavonoids
85	L-Tyrosine	C00082	8.4652	-0.70851	Amino acid	Organic acids and derivatives	Carboxylic acids and derivatives	Amino acids, peptides, and analogues
86	Dihydromyricetin	C02906	8.5528	-0.72162	Lipid	NULL	NULL	NULL
87	Diosmin	C10039	10.282	9.4964	Lipid	Phenylpropanoids and polyketides	Flavonoids	Flavonoid glycosides
88	L-Theanine	C01047	11.359	-0.78276	Xenobiotics	Organic acids and derivatives	Carboxylic acids and derivatives	Amino acids, peptides, and analogues
89	N-Methyl-L-glutamic acid	C01046	-15.598	-16.246	Energy	Organic acids and derivatives	NULL	NULL
90	Isobutyric acid	C02632	-18.463	-15.257	Lipid	Organic acids and derivatives	Carboxylic acids and derivatives	Carboxylic acids
91	Palmitic acid	C00249	-14.053	-15.199	Lipid	Lipids and lipid-like molecules	Fatty Acyls	Fatty acids and conjugates
92	4-Methylbenzoic acid	C01454	-13.615	-14.26	Xenobiotics	Benzenoids	Benzene and substituted derivatives	Benzoic acids and derivatives
93	Orientin	C10114	-9.9542	-11.895	Lipid	Phenylpropanoids and polyketides	Flavonoids	Flavonoid glycosides

94	Aminocaproic acid	C02378	-5.063	-8.2842	Xenobiotics	Lipids and lipid-like molecules	Fatty Acyls	Fatty acids and conjugates
95	Epigallocatechin gallate	C09731	-0.82141	-4.3764	Lipid	Phenylpropanoids and polyketides	Flavonoids	Flavans
96	2-Hydroxy-3-(4-hydroxyphenyl)propanoic acid	C03672	-2.3525	-3.6729	Amino acid	Phenylpropanoids and polyketides？	NULL	NULL
97	12-Hydroxydodecanoic acid	C08317	-0.82827	-3.5828	Lipid	Organic acids and derivatives	Hydroxy acids and derivatives	Medium-chain hydroxy acids and derivatives
98	Dihydrothymine	C00906	-0.65058	-2.9727	Nucleotide	Organoheterocyclic compounds	Diazines	Pyrimidines and pyrimidine derivatives
99	Biochanin A	C00814	-0.67578	-2.6434	Lipid	Phenylpropanoids and polyketides	Isoflavonoids	O-methylated isoflavonoids
100	m-Cresol	C01467	-0.82919	-2.3727	Xenobiotics	Benzenoids	Phenols	Cresols
101	2-Ketohexanoic acid	C00902	-3.9234	-2.2924	Lipid	Organic acids and derivatives	Keto acids and derivatives	Medium-chain keto acids and derivatives
102	Theobromine	C07480	3.5718	-2.1001	Xenobiotics	Organoheterocyclic compounds	Imidazopyrimidines	Purines and purine derivatives
103	1-Methylhistidine	C01152	-1.9845	-2.0456	Amino acid	Organic acids and derivatives	Carboxylic acids and derivatives	Amino acids, peptides, and analogues
104	N,N-Diethyl-m-toluamide	C10935	-2.2036	-1.9958	Xenobiotics	Benzenoids	Pesticides/Herbicides	NULL
105	4-Fluoro-L-threonine	C15533	-2.2522	-1.9288	Unknow	NULL	NULL	NULL
106	(2E)-Decenoyl-ACP	C03969	-0.63121	-1.7374	Amino acid	Organic acids and derivatives	Carboxylic acids and derivatives	Amino acids, peptides, and analogues
107	Adipate semialdehyde	C06102	0.47689	-1.6429	Xenobiotics	Lipids and lipid-like molecules	Fatty Acyls	Fatty acids and conjugates
108	Ethylmethylacetic acid	C18319	-0.84742	-1.5764	Lipid	Lipids and lipid-like	Fatty Acyls	Fatty acids and conjugates

						molecules		
109	Piperidine	C01746	1.2392	-1.3549	Unknow	Organoheterocyclic compounds	Piperidines	NULL
110	Azelaic acid	C08261	0.84259	-1.3387	Lipid	Lipids and lipid-like molecules	Fatty Acyls	Fatty acids and conjugates
111	Pyroglutamic acid	C01879	3.3687	-1.3235	Amino acid	Organic acids and derivatives	Carboxylic acids and derivatives	Amino acids, peptides, and analogues
112	Maleic acid	C01384	-0.79273	-1.2778	Carbohydrate	Organic acids and derivatives	NULL	NULL
113	2-Keto-6-acetamidocaproa te	C05548	3.2874	-1.1291	Amino acid	Organic acids and derivatives	Keto acids and derivatives	Medium-chain keto acids and derivatives
114	beta-Alanine	C00099	0.58012	1.2876	Amino acid	Organic acids and derivatives	Carboxylic acids and derivatives	Amino acids, peptides, and analogues
115	Leucine	C16439	0.64837	1.9677	Unknow	Organic acids and derivatives	Carboxylic acids and derivatives	Amino acids, peptides, and analogues
116	2-Methylbenzoic acid	C07215	0.41035	2.1813	Xenobiotics	Benzenoids	Benzene and substituted derivatives	Benzoic acids and derivatives
117	Prostaglandin G2	C05956	1.0957	2.2029	Lipid	Lipids and lipid-like molecules	Fatty Acyls	Eicosanoids
118	3-Methyl-2-oxovaleric acid	C03465	0.46073	2.3628	Amino acid	Organic acids and derivatives	Keto acids and derivatives	Short-chain keto acids and derivatives
119	trans-1,2-Cyclohexanediol	C03739	0.65942	2.4717	Xenobiotics	Organic oxygen compounds ?	NULL	NULL
120	Apiforol	C12124	-2.1683	2.5211	Lipid	NULL	Benzene and substituted derivatives	1-benzopyrans
121	Pimelic acid	C02656	1.3513	2.7548	Cofactors and	Lipids and lipid-like	Fatty Acyls	Fatty acids and conjugates

					Vitamins	molecules		
122	4-Quinolinecarboxylic acid	C06414	2.2482	2.9637	Unknow	Organoheterocyclic compounds	NULL	NULL
123	epsilon-Caprolactone	C01880	1.0841	3.0432	Xenobiotics	Organoheterocyclic compounds	Lactones	NULL
124	Eriodictyol	C05631	-11.566	3.0505	Lipid	Phenylpropanoids and polyketides	Flavonoids	Flavans
125	4-O-beta-D-Glucosyl-4-hyd roxycinnamate	C04415	1.0917	3.2485	Unknow	NULL	Benzene and substituted derivatives	Alcohols and polyols
126	Hydroxykynurenine	C02794	1.8981	3.3037	Amino acid	Organic oxygen compounds	Organooxygen compounds	Carbonyl compounds
127	Nororientaline	C05317	0.95481	3.5858	Unknow	NULL	Azacyclic compounds	1-hydroxy-2-unsubstitute d benzenoids
128	2-Methoxyestradiol	C05302	-1.7008	3.9119	Lipid	NULL	Steroids and steroid derivatives	Estrane steroids
129	Indolepyruvate	C00331	2.2092	3.9242	Amino acid	Organoheterocyclic compounds	Indoles and derivatives	Indolyl carboxylic acids and derivatives
130	Genipin	C09780	0.47639	4.2533	Unknow	Lipids and lipid-like molecules	Prenol lipids	Monoterpenoids
131	Phosphohydroxypyruvic acid	C03232	1.0636	4.4026	Amino acid	Organic oxygen compounds	Organooxygen compounds	Carbonyl compounds
132	Diosmetin	C10038	0.55503	5.6928	Lipid	Phenylpropanoids and polyketides	Flavonoids	O-methylated flavonoids
133	Carnosol	C09069	0.65914	6.0944	Unknow	Lipids and lipid-like molecules	Prenol lipids	Terpene lactones
134	Adipate	C06104	3.0377	6.1509	Xenobiotics	Lipids and lipid-like	Fatty Acyls	Fatty acids and conjugates

molecules
